# Veterinary Legislation

**Published:** 1891-06

**Authors:** 


### EDITORIAL DEPARTMENT.

### VETERINARY LEGISLATION, NEW YORK STATE.

It is an old aphorism, "in time of peace, prepare for war." The Veterinarians of New York State, have just been defeated at Albany, in an attempt at legislation for the benefit of themselves and the community. An act to establish a State Board of Veterinary Examiners, and to regulate the practice of Veterinary Medicine and Surgery throughout the State of New York (see appendix), was reported favorably from the General Laws' Committee, passed second reading, and was lost on final passage in the Assembly; it received 46 votes, out of about 100. A prominent Veterinarian tells us that, "its defeat was largely due to the fact, that prominent papers in the State opposed the measure; certain papers criticised the bill, in an ignorant way, showing, that they knew nothing of its purpose." This may be true, but if it is the only cause, it shows lack of organization and discipline on the part of the Veterinarians. We have always found that the prominent newspapers, are willing to advance the interests of our profession, and if they show ignorance of the foundation of our cause, they should have been instructed and convinced of its merits. Professional men have not much familiarity with the cunning ways, and shrewd deeds of the politician. The political profession is one *per se,* which requires as long an elementary study, as any other, and for which, new matters must be presented in a very concise, clear form.

Now is the time for action, let us at once study the merits of the act, which has just been defeated. In the JOURNAL, during the last year, will be found copies of the Bills, which have been presented and passed in Pennsylvania, New Jersey, and California. Let us study those ; let every Veterinarian in New York State, see his Representative at once, go over the act with him, and inquire wherein it was defective, or why there was any serious objection to it. Find out if the Representative will object to the Bill if re-elected, or if he will support it, and find out what changes will be advantageous to it; do not delay in this matter for a month, or until next week, but see the MEMBER at once, and immediately, having seen him, report the result of it, to the Secretary of the New York State Veterinary Medical Society. When the next election takes place, let every Veterinarian see the candidates, interview them, and support the one, who will promise to support proper legislation for the Veterinary profession. If everyone will do his duty at once, and make a report, these can be properly digested before the next meeting of the New York State Veterinary Medical Society, and reported in such form, that a more suitable Bill, if such is required, can be prepared, and be already worked up before the meeting of the legislature. We assure the Veterinarians of New York State, that our pages will be freely open at all times for the advancement of this matter, and we trust, that they will use them for an expression of their opinions. Remember that it is important to see other Veterinarians, who are not members of the New York State Society, to learn their views, and if they are in opposition to persuade them into friendship.

### AN ACT

To establish a State Board of Veterinary Examiners and to regulate the practice of Veterinary Medicine and Surgery throughout the State of New York.

The People of the State of New York, represented in Senate and Assembly, do enact as follows :

SECTION 1. Within sixty days after the passage of this act, the Governor of the State shall appoint seven Veterinarians each of whom shall hold a certificate of graduation from an incorporated Veterinary College or University, to be selected from a number not exceeding fourteen (14) as follows: Seven nominated by the New York State Veterinary Medical Society, and seven by the Long Island Veterinary Medical Society, and their successors shall be appointed in like manner.

SECTION 2. Each member shall take and file the constitutional oath of office required of public officers.

SECTION 3. The said members of said Boad shall meet on the second Tuesday of July, in the year 1891, in the city of Syracuse for organizatisn. The hour and place of meeting to be designated by the Secretary of the State Society, at which time they shall elect a president, secretary and treasurer, and may adopt such rules and regulations not inconsistent with law as they may deem necessary.

SECTION 4. Each member of said Board shall hold office during good behavior and may be removed by the Governor for mis-conduct or incompetency upon reasonable notice of charges made against him and an opportunity to be heard. But no member shall hold office after he is 65 years of age, and the term of office of any member shall expire upon his reaching that age.

SECTION 5. It shall be the duty of said Board to examine any person over 21 years of age and a resident of this State applying for licenses under this act, provided that said applicant shall first show to the satisfaction of said Board that he has practiced Veterinary Medicine and Surgery for a period of not less than ten years, or that said applicant holds a diploma or certificate of practice from an incorporated Veterinary College or University, or from the Agricultural Department of Cornell University, after at least two years of continuous study thereat, and shall have duly paid to the treasurer of said Board all fees hereinafter prescribed.

SECTION 6. Said Board shall keep a record of all persons licensed by them to practice Veterinary Medicine and Surgery, and shall file with the Secretary of State and with the Secretary of the New York State Veterinary Medical Society, on or before the first day of July of each year a detailed report of the proceediugs of said Board together with a statement of all their receipts and disbursements.

The members of said Board shall hold at least one meeting each year for the examination of applicants and as many other meetings as they may deem requisite. The first meeting of the said Board shall be held on the first Tuesday of May in the city of Syracuse, of which proper and timely notice shall be given through the Veterinary Journals of the State.

ARTICLE 5. Five members of said Board shall constitute a quorum, and the concurring vote of a majority of the members present at a meeting at which there is a quorum shall be deemed the decision of the Board.

Article 6. Every applicant for a license shall upon making his application pay to the Board of Examiners the sum of twenty dollars, and on receiving his certificate the further sum of five dollars. In case of failure at any such examination the applicant after the expiration of six months and within one year shall have the privilege of a second examination by the Board or by a committee of one or more of the Board without the payment of an additional application fee.

Article 7. From the income provided by this Act the expenses of the examiners shall be paid. Any surplus after all proper expenses incurred by the Board shall have been met, if any surplus shall remain, shall be apportioned among said examiners pro-rata, as compensation for their services.

After July 1st. 1891, every person now practicing Veterinary Medicine and Surgery in this State by virtue of Chapter 313 of the Laws of 1886, who does not hold a certificate of graduation from some incorporated Veterinary College or University or the Agricultural Department of Cornell University shall assume the title of "Farrier" until he shall obtain a certificate of graduation from an incorporated Veterinary College or the Agricultural Department of Cornell University, or a license to practice from the State Board of Examiners. And it shall be the duty of the County Clerk to write opposite the title the record of the Veterinarians, Veterinary Surgeon, or any other like title of the name of every practioner who has registered under the provisions of Chapter 313 of the Laws of 1886, and who does not before July 1st. 1891, produce a diploma as evidence of his graduation from some incorporated Veterinary College or University the words the title annulled and the title "Farrier" substituted under the provisions of Chapter of the Laws of 1891, inserting the Chapter under this Act.

After July 1st. 18891, it shall be unlawful for any person to practice Veterinary Medicine and Surgery or any branch thereof in this State who are not now legally authorized to practice and those who shall not obtain the certificate of qualification after due examination from said Boad of Examiners.

Nothing in this Act shall be construed to prohibit students from prescribing under the supervision of duly authorized preceptors or to prohibit gratuitous services in cases of emergency or to prohibit any legally qualified practitioner, residing in another State, meeting Surgeons of this State in consultation or residing on the border of a neighboring State and duly authorized under the law thereof to practice Veterinary Medicine and Surgery therein whose practice extends into the limits of this State providing that such practitioner shall not open an office, or appoint a place to meet and treat patients, or receive calls within the limits of the State of New York.

Section 7. Every violation of this Act shall be deemed a misdemeanor.

Section 8. This Act shall take effect immediately.

This bill was introduced in the Legislature Feb. 26th, by Mr. Peck, of Cortland County.

